# Digital Health Literacy Questionnaire for Older Adults: Instrument Development and Validation Study

**DOI:** 10.2196/64193

**Published:** 2025-03-19

**Authors:** Xinxin Wang, Chengrui Zhang, Yue Qi, Ying Xing, Yawen Liu, Jiayi Sun, Wei Luan

**Affiliations:** 1 Shuguang Hospital Affiliated to Shanghai University of Traditional Chinese Medicine Shanghai China; 2 Shanghai Jiao Tong University School of Nursing Shanghai China; 3 Department of Social Medicine School of Health Management Harbin Medical University Harbin China; 4 Shuguang Clinical Medical College Shanghai University of Traditional Chinese Medicine Shanghai China

**Keywords:** digital health literacy, digital literacy, older adults, instruments, reliability, psychological measures, questionnaire, China

## Abstract

**Background:**

The integration of digital technology into older adult health and care has enhanced the intelligence of health and older adult care products and services while also transforming how seniors acquire and share health information. Assessing older adults’ digital health literacy (DHL) is crucial for developing targeted interventions.

**Objective:**

This study aims to develop and validate a DHL assessment questionnaire for older adults. It also seeks to evaluate the questionnaire’s scientific validity and feasibility among community-dwelling older adults in China, providing a reliable tool for assessing their level of DHL.

**Methods:**

A literature review, focus group discussions, and the Delphi method were used to construct the questionnaire item pool and perform item screening. Item analysis was conducted for comprehensive evaluation, and questionnaire validity was assessed through construct validity (exploratory factor analysis, confirmatory factor analysis, convergent validity, and discriminant validity), content validity, and criterion-related validity. Reliability was analyzed using Cronbach alpha coefficient, split-half reliability, and test-retest reliability.

**Results:**

The study included 710 participants. Item analysis indicated that the questionnaire had strong discriminant validity. Correlation coefficient analysis showed that the item-total correlation coefficients ranged from 0.497 to 0.920 (P<.01). After multiple exploratory factor analyses, 6 common factors were extracted, with a cumulative variance contribution rate of 73.745%. Confirmatory factor analysis demonstrated a good model fit (χ^2^/df=2.803, root-mean-square error of approximation=0.071, comparative fit index=0.907, goodness-of-fit index=0.773, incremental fit index=0.908, Tucker-Lewis index=0.901, normed fit index=0.863). The questionnaire demonstrated favorable convergent validity, content validity, and criterion-related validity. The total Cronbach α coefficient was 0.976, with dimension-specific Cronbach α coefficients ranging from 0.819 to 0.952, indicating satisfactory internal consistency. Additionally, the test-retest reliability coefficient for the total questionnaire was 0.925, demonstrating good stability over time.

**Conclusions:**

This study developed a questionnaire specifically designed to assess DHL in older adults through a scientifically rigorous and systematic process. The questionnaire demonstrates strong psychometric properties and can serve as an empirical tool for health professionals to design personalized intervention policies and enhance health service delivery.

## Introduction

Health literacy (HL) is the ability of an individual to access, understand, evaluate, and apply health information to make appropriate health decisions. It can help people make rational judgments about health care, disease prevention, and health promotion in their daily lives, thereby maintaining or enhancing an individual’s quality of life throughout the life span [1]. Digital health literacy (DHL) is an expansion of HL in the digital era, which refers to an individual’s ability to access, process, communicate, and understand health information and services in the context of the use of digital technology and information to promote and improve individual and collective health through effective health decision-making [2]. Different from HL, DHL emphasizes the technical skills required in the digital environment, such as using digital devices and the internet to search for health information, operating health-related apps, identifying and assessing the reliability of online health information, safeguarding the privacy and security of personal health information, and communicating effectively on digital platforms. In addition, DHL involves using critical thinking in digital environments, using digital tools for self-management and health monitoring, and adapting learning to the development of digital technology and health information, which are not included in the concept of HL [2,3]. Studies have shown significant associations between DHL and anxiety [4,5], health-promoting behaviors [6], self-efficacy [6,7], and self-care competence [7] in the older adult population. After controlling for sociodemographic variables, DHL in older adults was found to be able to directly and positively influence health-related quality of life in the study by Liu et al [8]. Kim et al [9] stated that there is a positive association between DHL and health-related behaviors. DHL in older adults significantly promotes health behaviors, health support behaviors, and disease management behaviors. Arcury et al [10] found a positive correlation between DHL in older adults and individual health knowledge, attitude, and computer use.

In recent years, digital technology has developed rapidly and has been increasingly applied to the field of older adults’ health and aging. This advancement has enhanced the intelligence of health and aging products while also requiring a higher level of DHL for older adults to adapt to society and promote health [11]. To date, 5 DHL evaluation tools have been used for older adults [3]. Among them, the eHealth Literacy Scale (eHEALS) [12] is the most widely used DHL assessment tool, primarily measuring an individual’s self-perceived ability to use information technology to process health information. However, it has a relatively limited scope of skills for assessment. By contrast, the electronic Health Literacy Scale [13] and the Digital Health Literacy Instrument [14] focus on evaluating individuals’ operational skills in gathering online health information. However, these 2 tools may only partially cover the dimensions of DHL, particularly the practical skills required for operating digital devices and interactive communication. While the eHealth Literacy Questionnaire [15] was designed to keep pace with the growing prevalence of digital technology, it does not sufficiently address the security of personal information, content creation, and information exchange. The Digital Health Literacy Assessment [16] scale primarily focuses on using computers, smartphones, and other devices to access and assess health information. However, it falls short of accommodating the rapidly evolving demands of digital health capabilities. The emergence of new digital technologies may extend beyond the scope of assessment in existing DHL assessment tools. If assessment tools fail to accurately measure older adults’ DHL levels, they may struggle to effectively use digital health information resources and fully engage with digital tools for health management. This could lead to deficiencies in understanding health issues, difficulties in making informed health decisions, and an increased risk of health inequalities among older adults.

The Digital Competence Framework (DIGCOMP) was developed by the European Commission’s Joint Research Centre in 2013 to identify and define digital competencies relevant to all citizens living and working in Europe. The framework outlines 5 key competence areas: information, communication, content creation, safety, and problem-solving [17]. The framework provides a comprehensive and systematic approach to assessing digital competence, enabling comparisons across regions and groups. It also categorizes different levels of digital competence and encourages individuals to engage in lifelong learning to continuously refine and enhance their digital skills. Developed through extensive research and practice, DIGCOMP encompasses a range of digital competencies at different levels, from basic to advanced, allowing assessment tools to align with older adults’ varying skill levels. Additionally, the DIGCOMP framework covers various aspects of digital competence and can be adapted to meet the specific needs and real-life situations of older adults, ensuring better alignment with their characteristics. Studies have been conducted to develop and validate assessment questionnaires for the everyday digital literacy of older adults based on the DIGCOMP framework, demonstrating good psychometric properties. These questionnaires have been successfully used to assess the digital literacy of older adults in Korea [18].

Because of declining cognitive function and changes in health status, older adults have an increasing need for health information and services. As the digital health domain continues to evolve, health management processes and efficiencies are being optimized. However, this also presents the challenge of a digital divide for older adults. Differences in educational background and technology acceptance lead to significant variations in their digital health capabilities [19]. Therefore, accurately assessing the digital health competence of older adults and implementing targeted interventions are crucial for improving their DHL. Currently, no digital health assessment tools specifically designed for older adults have been developed. In this context, our study constructed a questionnaire to assess DHL in older adults, using DIGCOMP as a theoretical framework while considering their cognitive and physiological characteristics. This questionnaire aims to identify the current level of DHL among older adults, providing a scientific basis for health care professionals to develop personalized interventions and health management policies, as well as to optimize health service processes.

## Methods

### Building DHL Questionnaire Entries for Older Adults

The research group conducted a scoping review to comprehensively gather relevant research findings on DHL evaluation questionnaires for older adults [3]. The initial literature search was performed in January 2022, followed by a supplementary search in June 2022. After the screening process, 16 published articles were identified that included evaluations of DHL in older adults, encompassing 5 assessment tools. Our analysis revealed that previous studies on DHL among older adults have primarily focused on assessing their ability to access, evaluate, and apply health information, while lacking assessments of their ability to communicate, integrate health information, and protect their privacy. Furthermore, no assessment tools specifically designed to address the unique characteristics of the older population have yet emerged. Based on these findings, we developed a new questionnaire with particular emphasis on the above dimensions, considering the cognitive and operational characteristics of the older population. Semistructured interviews were conducted before expert consultation (Multimedia Appendix 1). The corresponding author (WL) recruited experts through her personal network and applied a snowball sampling method to further expand the participant pool. The criteria for selecting experts for the semistructured interviews are listed in Textbox 1.

**Criteria for selecting experts for the semistructured interviews.**

1. Health management of older adults
  - Experts were required to have over 10 years of experience in the field, hold at least an intermediate professional title, possess a postgraduate degree or higher, voluntarily participate, and be able to complete consultations in a timely manner.
2. Electronic health and digital health
  - Experts were required to have more than 5 years of experience, hold at least an intermediate professional title, have a bachelor’s degree or higher, voluntarily participate, and be able to complete consultations in a timely manner.

A total of 6 experts participated in interviews and group discussions. These included a PhD supervisor specializing in health management, a chief physician in community general medicine, a chief physician in geriatric medicine, a master’s supervisor specializing in public health, a master’s supervisor in geriatric nursing, and a chief nurse. The interview outline included the following questions:

- What do you think should be included in the evaluation index of DHL for older adults?
- What fields of expertise do you think should be consulted via correspondence, and for what reasons?

The original DHL questionnaire was developed based on expert interviews. Five rounds of group discussions were conducted to refine the questionnaire, ensuring its alignment with China’s national context and cultural background. Subsequently, the first version of the DHL Questionnaire was formulated, using DIGCOMP as its theoretical framework.

### Validation of Questionnaire Contents

This study used expert consultation to validate the questionnaire’s content. Sixteen experts from Shanghai’s tertiary hospitals, colleges, universities, and technology companies—who have long been engaged in geriatric care, geriatrics, internet and health care, health policy, and computing—were selected for this study. The criteria for selecting experts were the same as those used for the semistructured interviews; however, the experts themselves were different. The experts’ questionnaire consisted of 3 parts (Textbox 2).

**Parts of the experts’ questionnaire.**

1. Introduction
  - Outlining the purpose of the study, instructions for completing the questionnaire, details on how and when to submit it, contact information, and acknowledgments.
2. Digital health literacy evaluation questionnaire correspondence form for older adults
  - Experts rated the importance and relevance of each dimension and item on a scale of 1 to 5 (ranging from “very unimportant” to “very important”) and 1 to 4 (ranging from “very irrelevant” to “very relevant”), with 2 additional columns—“Opinions and Reasons for Indicator Modification or Deletion” and “Items to be Added”—for experts to provide modification suggestions.
3. General information and expert authority questionnaire
  - Collecting basic information about the experts, along with their self-assessments of their authority and familiarity with the questionnaire content.

Three rounds of expert correspondence were conducted, with questionnaires distributed and returned via WeChat (Tencent Holdings Limited) or email. Upon receipt, the research team checked for completeness and verified any ambiguities or omissions with the experts to ensure the validity of the responses. The inclusion criteria for the entries were as follows: a mean importance score >3.5, a total score rate >20%, and a coefficient of variation <0.25 [20]. Entries were adjusted based on discussions within the research team, incorporating expert modifications. After each round, responses were compiled, and items with significant opinion divergences were identified. In the subsequent round, these contentious items, along with summaries of expert opinions (median and standard deviation), were presented to encourage reflection and adjustment, gradually moving toward consensus. The study used Kendall *W* to measure the consistency of evaluations among experts, with the consultation stopping when Kendall *W* ranged between 0.4 and 0.5, indicating a satisfactory level of consensus [21]. The final DHL questionnaire for older adults included 6 dimensions and 46 items.

### Evaluation of the Reliability and Validity of the Questionnaire

#### Participants and Data Collection

Given the high acceptance and response rate among older adults following advocacy by community health workers, and the fact that those able to attend the physical examination in person generally met the study’s inclusion criteria, convenience sampling was used. The inclusion criteria were as follows: (1) signed informed consent and willingness to participate in the research; (2) aged 60 years or older and residing in the community for over 6 months; (3) possession of full communicative language abilities, including listening comprehension, oral expression, reading comprehension, and written expression. The exclusion criteria were (1) diagnosed mental disorders (eg, schizophrenia, delusions) or cognitive disorders (eg, Alzheimer disease, vascular cognitive impairment) and (2) diagnosis of a major or advanced terminal illness, such as a malignant tumor or end-stage renal disease.

Our study utilized an online survey method, with uniformly trained investigators rigorously selecting participants based on the inclusion and exclusion criteria. After eligible older adults completed their physical examination, the investigator explained the purpose and significance of the study. Participants were guided on how to navigate the questionnaire interface, key considerations for completing the questionnaire, and the rules for successful submission. Older adults completed the questionnaire independently on the spot. To prevent duplicate participation, the questionnaire was configured to allow only 1 submission per WeChat user. After the survey concluded, researchers conducted a quality check on all questionnaires. A preexperiment revealed that it was nearly impossible for older adults to complete the questionnaire within 2 minutes while carefully reading each question. Therefore, to ensure data quality, questionnaires completed in under 2 minutes were excluded.

The sample size was determined based on the principle that factor analysis requires at least 5-10 times the number of questionnaire items [22]. Given that the questionnaire contains 46 items and considering a 20% increase for potential invalid responses, the sample size was calculated as 46 × 5 × (1 + 20%) = 276. As both exploratory and confirmatory factor analyses require 2 independent samples, the minimum required sample size for the study was 552. Study participants were selected from August to November 2023 among 710 older adults undergoing their annual physical examination in Shanghai. The obtained samples were randomly divided into 2 groups for pretesting (n=355) and formal testing (n=355).

#### Ethics Approval

The study was approved by the Ethics Committee of Renji Hospital, affiliated with Shanghai Jiao Tong University School of Medicine (approval number RA-2021-465). Participants understood the purpose, process, risks, and benefits of the study and voluntarily agreed to participate. The survey adhered to the principles of anonymity and confidentiality, and responses were collected anonymously. Health science popularization books were provided to participants as compensation.

#### Instruments

In our study, the questionnaire was in Chinese and consisted of 4 parts (Multimedia Appendix 2): (1) informed consent form; (2) general information questionnaire (designed by the researcher), which included information on gender, age, education level, marital status, residential situation, household monthly income, the primary source of income, and medical payment methods; (3) the eHEALS (developed by Norman et al [12]), which consists 8 questions assessing the ability to apply online health information and services, judgment ability, and decision-making ability. It is scored on a 5-point Likert scale, with a total score ranging from 8 to 40, where higher scores indicate better eHealth literacy (eHL). The scale demonstrated high internal consistency (α=.967); (4) Digital Health Literacy Questionnaire for Older Adults (designed by researchers WL and XW), which includes 6 dimensions: Information (14 items), Interaction (11 items), Content (3 items), Safety (7 items), Attitude (4 items), and Behavior (7 items). A 5-point Likert scale was used, with each item scored from 1 to 5, ranging from “very inconsistent” to “very consistent,” where higher scores indicate better DHL.

### Statistical Analysis

Excel 2019 (Microsoft Corporation) was used for raw data entry, and SPSS 25.0 (IBM Corp.) and AMOS 26.0 (IBM Corp.) statistical software were used for statistical analysis. Sociodemographic information was expressed as the number of cases and percentages. Given that all variables in our study were bivariate normally distributed and exhibited a linear relationship, we used Pearson correlation analysis.

The first pretest sample (N=355) was subjected to item analysis, exploratory factor analysis (EFA), and further screening of entries to develop the formal questionnaire. The questionnaire items were comprehensively evaluated based on the item analysis screening criteria listed in Textbox 3.

**Item analysis screening criteria.**

1. Critical ratio method
  - The total questionnaire score was divided into high and low groups, comprising the top 27% and bottom 27%, respectively. The critical value (*t* value) for each item between these groups was compared, with a requirement of *t*≥3 and a statistically significant difference.
2. Correlation coefficient method
  - The correlation coefficient between each item and the total score, as well as the corrected item-total correlation coefficient, should be ≥0.4.
3. Homogeneity test
  - The Cronbach α coefficient of the total questionnaire should not increase after deleting any item. The factor loading of each item on the common factors of the questionnaire should be ≥0.4, and the communality should be ≥0.2. An item was deleted if it failed to meet at least three of these 6 indicators [23].

The Kaiser-Meyer-Olkin (KMO) test and Bartlett test of sphericity were used to assess the adequacy of the data. A KMO value close to 1 and *P*<.05 after the Bartlett test indicate that the original variables are suitable for factor analysis. Principal component analysis was used to extract common factors with eigenvalues greater than 1, combined with the scree plot for judgment. The varimax method of orthogonal rotation was applied to enhance factor loadings, with strong associations considered to exist when factor loadings were ≥0.4. If an item had factor loadings ≥0.4 across multiple dimensions, it was assigned to the dimension with the highest factor loading [24]. The deletion criteria were as follows: (1) items with loadings <0.4 on their respective factors; (2) items with communalities <0.2; (3) items with multiple loadings of similar values across dimensions; (4) items with factor loadings inconsistent with the original conceptual framework and inexplicable; and (5) dimensions with fewer than 3 items [25].

Validation factor analysis, convergent and discriminant validity tests, content validity tests, validity scale assessments, and internal consistency reliability tests were conducted on the second round of formal questionnaire test samples (N=355) to verify the reliability and validity of the questionnaire.

To further verify the structural validity of the questionnaire, confirmatory factor analysis was conducted using the maximum likelihood method to estimate model parameters. The chi-square to degrees of freedom ratio (*χ*^2^/*df*), root-mean-square error of approximation, goodness-of-fit index, adjusted goodness-of-fit index, comparative fit index, incremental fit index, Tucker-Lewis index, and normed fit index were used to evaluate the model [26]. When the average variance extracted (AVE) is greater than 0.5 and the composite reliability exceeds 0.7, the questionnaire demonstrates ideal convergent validity. Additionally, when the correlation between questionnaire dimensions is significant but remains lower than the square root of the corresponding AVE, the questionnaire exhibits desirable discriminant validity [27].

In this study, experts from relevant fields (N=16) were invited to evaluate the content validity of the questionnaire. The item-level content validity index (I-CVI) represents the proportion of experts who rated each item as 3 or 4. The scale-level content validity index (S-CVI/Ave) is the average of all I-CVIs. When the number of experts exceeds 6, an I-CVI>0.78 and an S-CVI/Ave>0.90 are generally considered to indicate good relevance of the items and questionnaire to the conceptual content being measured [28].

The eHEALS was used as the criterion tool in this study. A positive and statistically significant correlation between the criterion tool and the DHL questionnaire for older adults, both in total score and across dimensions, indicates that the criterion-related validity is satisfied [29].

Cronbach α coefficient and Spearman-Brown split-half reliability are the most commonly used methods for testing the internal consistency reliability of questionnaires. Generally, a Cronbach α coefficient greater than 0.500 for each dimension and a total Cronbach α coefficient and Spearman-Brown split-half reliability greater than 0.700 indicate acceptable reliability. Retest reliability analyses were conducted with a retest sample (N=20) [29]. For all tests, the significance level was set at α=.05.

## Results

### Characteristics of Participants

The study included 710 participants, consisting of 354 males (49.9%) and 356 females (50.1%). Participants ranged in age from 65 to 100 years, with a mean age of 70.7 (SD 6.3) years. The results are presented in Table 1.

**Table 1:** Participant characteristics (N=710).

Variables			Group 1 (N=355), n (%)		Group 2 (N=355), n (%)		Between groups 1 and 2				
							Chi-square (*df*)		*P* value		
**Gender**							0.09 (1)		.77		
	Male	179 (50.4)		175 (49.3)							
	Female	176 (49.6)		180 (50.7)							
**Age**							3.529 (3)		.32		
	60-65	210 (59.2)		231 (65.1)							
	66-70	83 (23.4)		78 (22.0)							
	71-75	30 (8.5)		22 (6.2)							
	＞76	32 (9.0)		24 (6.8)							
**Educational level**							3.772 (3)		.29		
	Junior high and below	188 (53.0)		164 (46.2)							
	High school/secondary vocational	126 (35.5)		138 (38.9)							
	College/university	37 (10.4)		47 (13.2)							
	Postgraduates	4 (1.1)		6 (1.7)							
**Marital status**							4.198 (2)		.12		
	Married	287 (80.8)		304 (85.6)							
	Divorce	24 (6.8)		13 (3.7)							
	Widow	44 (12.4)		38 (10.7)							
**Residential situation**							7.786 (4)		.10		
	Living alone	46 (13.0)		42 (11.8)							
	Living with spouse	146 (41.1)		172 (48.5)							
	Living with children	73 (20.6)		49 (13.8)							
	Living with spouse and children	86 (24.2)		90 (25.4)							
	Other	4 (1.1)		2 (0.6)							
**Household monthly income**							7.662 (3)		.054		
	<4000 CNY^a^	108 (30.4)		126 (35.5)							
	4001-6000 CNY	110 (31.0)		104 (29.3)							
	6001-8000 CNY	97 (27.3)		71 (20.0)							
	>8000 CNY	40 (11.3)		54 (15.2)							
**Primary source of income**							5.541 (3)		.14		
	Pension	306 (86.2)		323 (91.0)							
	Child support	27 (7.6)		22 (6.2)							
	Reemployment income	12 (3.4)		6 (1.7)							
	Other	10 (2.8)		4 (1.1)							
**Medical payment methods**							6.681 (3)		.08		
	Urban Medical Insurance	249 (70.1)		275 (77.5)							
	Rural Medical Insurance	66 (18.6)		42 (11.8)							
	Employee Medical Insurance	36 (10.1)		34 (9.6)							
	Other	4 (1.1)		4 (1.1)							
**Chronic disease**							6.613 (3)		.08		
	Without	62 (17.5)		58 (16.3)							
	1	157 (44.2)		135 (38.0)							
	2	88 (24.8)		90 (25.4)							
	≥3	48 (13.5)		72 (20.3)							

^a^1 CNY=US $0.14.

### Item Analysis

The results of the item analysis are summarized in Multimedia Appendix 3. In the extreme group comparisons, all items showed statistically significant differences between the high- and low-score groups (*P*<.001). Correlation coefficient analysis indicated that item-total correlation coefficients ranged from 0.468 to 0.744, while corrected item-total correlation coefficients ranged from 0.442 to 0.725 (*P*<.001). The Cronbach α coefficient for the total questionnaire was 0.981, and no increase was observed after deleting any item. Factor analysis revealed that item factor loadings ranged from 0.365 to 0.697, with communalities ranging from 0.446 to 0.677. Ultimately, 3 items failed to meet 1 criterion; however, they did not meet the deletion threshold. Therefore, all 46 items were retained for further analysis.

### Validity Analysis

#### Construct Validity

##### Exploratory Factor Analysis

EFA was conducted on the 46 items, yielding a KMO value of 0.955 and a significant Bartlett test of sphericity (*χ*^2^_1035_=8299.714, *P*<.001), indicating that the data were suitable for factor analysis. Six common factors with eigenvalues >1 were extracted, accounting for 53.810% of the cumulative variance. The scree plot also confirmed that retaining 6 factors was appropriate. EFA was performed multiple times, setting the number of factors to 6, and noncompliant items were gradually removed. Ultimately, 39 items remained, forming 6 factors with a cumulative variance contribution rate of 73.745%. The factor loadings for the items in each dimension ranged from 0.452 to 0.815, as detailed in Multimedia Appendix 4.

##### Confirmatory Factor Analysis

**Figure 1:**
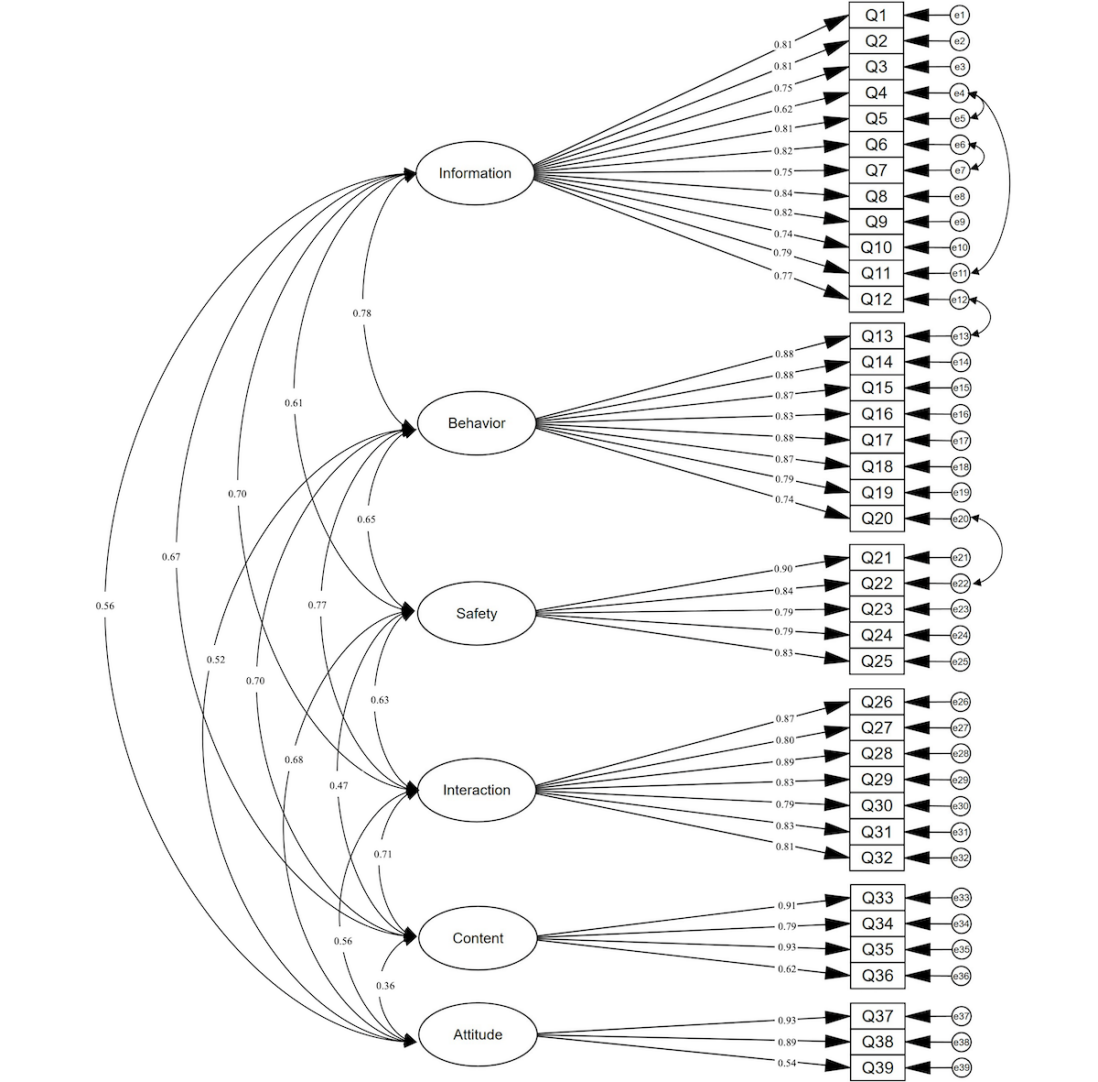
The confirmatory factor analysis model diagram.

The results indicated that the model was identifiable and successfully converged but initially exhibited poor goodness of fit. The modification indices revealed high correlations between 5 pairs of error terms, each with values greater than 10, suggesting potential local dependence issues in the model [26]. Upon further analysis, we found that while these item pairs were designed to measure distinct concepts, they exhibited some conceptual overlap. This finding was consistent with the high modification indices, indicating that the error terms might not be entirely independent as initially assumed. To address this, and after confirming that the model’s complexity was not artificially inflated, we sequentially added 5 error covariances to account for these observed relationships. Following these adjustments, the standardized regression coefficients of the revised model ranged from 0.538 to 0.931, indicating a good model fit. The model fit indices are presented in Table 2, and the confirmatory factor analysis model diagram is shown in Figure 1.

**Table 2:** Goodness-of-fit indices for confirmatory factor analysis of the digital health literacy questionnaire in older adults.

Item	Chi-square*/df*	Root-mean-square error of approximation	Comparative fit index	Goodness-of-fit index	Incremental fit index	Tucker-Lewis index	Normed fit index
Reference standard	<5	<0.08	>0.9	>0.9	>0.9	>0.9	>0.9
Initial model	3.404	0.082	0.875	0.733	0.876	0.855	0.833
Revised model	2.803	0.071	0.907	0.773	0.908	0.901	0.863

#### Convergent and Discriminant Validity

##### Convergent Validity

Based on the well-fitted model, the convergent validity of the questionnaire was tested, with results presented in Table 3. The factor loadings for the items across the 6 dimensions ranged from 0.538 to 0.931, all exceeding 0.500, indicating strong representativeness of the items within their respective dimensions. Additionally, the AVE for each dimension ranged from 0.608 to 0.768, all above the 0.500 threshold, while the composite reliability values ranged from 0.841 to 0.952, all exceeding 0.700, confirming the excellent convergent validity of the questionnaire.

**Table 3:** Convergent validity of the digital health literacy questionnaire in older adults.

Factors and items		Factor loadings	Average variance extracted	Composite reliability
**Information**			0.608	0.949
	1. I pay attention to whether health information is released and disseminated by official or authoritative institutions	0.814		
	2. I compare similar health information	0.809		
	3. I verify the correctness of health information from other sources	0.752		
	4. I remain vigilant about the health information I obtain and do not easily believe it	0.618		
	5. I understand digital health technologies (eg, wearable devices, smart health electronic products)	0.810		
	6. I understand that digital technologies can be used for health management or health promotion (such as health and medical mobile apps)	0.817		
	7. I do not immediately share health information with others after receiving it but first check the content	0.751		
	8. I can browse, search, and obtain health information through digital devices or software	0.844		
	9. I pay attention to updates on health information	0.818		
	10. I can judge whether health information is related to commercial interests (eg, contains product advertisements)	0.742		
	11. I understand that digital health devices or software can be used to store personal health information	0.788		
	12. I have used digital health devices or software to record personal health information	0.768		
**Behavior**			0.714	0.952
	13. I know how to use digital health tools to track my health behavior	0.880		
	14. I can judge whether digital health tools are trustworthy	0.879		
	15. During the use of digital health tools, I can adjust my frequency, intensity, and methods based on the actual situation	0.872		
	16. I am used to using digital services to handle health information	0.832		
	17. I can use digital devices or electronic health products or software	0.878		
	18. I know when, how, and what health information to use	0.874		
	19. If necessary, I think I can persist in using digital health tools	0.790		
	20. I believe the use of digital technologies is beneficial for my health management	0.743		
**Safety**			0.688	0.917
	21. I believe I have the right to pursue legal responsibility for unauthorized data acquisition or improper data storage that leads to data breaches	0.895		
	22. I believe I have ownership of personal data, and others can only obtain my personal health data with my authorization	0.840		
	23. I do not click on unsafe web links; I do not visit websites that are flagged as risky	0.787		
	24. I can avoid health risks related to the use of digital technologies that threaten physical and mental health	0.792		
	25. I know the potential security risks in the online environment	0.829		
**Interaction**			0.693	0.940
	26. I can share information with others on the internet	0.869		
	27. I can share the information I obtained online with others offline	0.803		
	28. I can use digital devices or software to communicate health information with others	0.894		
	29. I am familiar with the user interface of digital devices or software	0.829		
	30. I use information dissemination platforms (eg, Weibo, WeChat Moments) to share information	0.792		
	31. I can use digital devices or software to communicate health information with artificial intelligence	0.825		
	32. I imitate the health-promoting behaviors or health management methods mentioned in health information	0.811		
**Content**			0.768	0.908
	33. I can edit and improve health content created by myself or others	0.907		
	34. I can protect the integrity of original works and cite sources when referencing	0.787		
	35. I can integrate health information from multiple sources and rephrase it	0.929		
	36. In the past 12 months, I have participated in online health lectures and health care experience sharing activities	0.621		
**Attitude**			0.650	0.841
	37. I adhere to the correct political direction in online behavior	0.931		
	38. I do not fabricate or spread false, unverified health information	0.891		
	39. I care about health information related to myself	0.538		

##### Discriminant Validity

Further examination of discriminant validity showed that all questionnaire dimensions were significantly correlated, with correlation coefficients ranging from 0.363 to 0.779 (*P*<.001). These values were all lower than the square roots of the corresponding AVEs, indicating that while the dimensions are interrelated, they maintain sufficient discriminant validity. This confirms that the questionnaire structure demonstrates ideal discriminant validity. See Table 4 for details.

**Table 4:** Discriminant validity of the digital health literacy questionnaire in older adults.

Dimensions	Information	Behavior	Safety	Interaction	Content	Attitude
Information	1	—^a^	—	—	—	—
Behavior	0.779^b^	1	—	—	—	—
Safety	0.614^b^	0.650^b^	1	—	—	—
Interaction	0.704^b^	0.773^b^	0.629^b^	1	—	—
Content	0.660^b^	0.704^b^	0.465^b^	0.708^b^	1	—
Attitude	0.555^b^	0.518^b^	0.676^b^	0.560^b^	0.363^b^	1
Square root of average variance extracted	0.779	0.845	0.829	0.832	0.876	0.806

^a^Not applicable.

^b^*P*<.01.

#### Content Validity

Based on expert ratings, the I-CVI of the questionnaire ranged from 0.81 to 1.000 and S-CVI/Ave was 0.92, both meeting the required standards, indicating good content validity.

#### Criterion-Related Validity

The criterion validity analysis showed that the total and dimension scores of the Digital Health Literacy Questionnaire for Older Adults were positively correlated with the total and dimension scores of the eHEALS, with correlation coefficients ranging from 0.363 to 0.980, all statistically significant (*P*<.01). This demonstrates good criterion-related validity of the questionnaire, as shown in Multimedia Appendix 5.

### Reliability Analysis

#### Cronbach α Coefficient

The results showed that the total questionnaire’s Cronbach α coefficient was 0.976, and each dimension’s Cronbach α coefficient ranged from 0.819 to 0.952, demonstrating high internal consistency of the questionnaire.

#### Split-Half Reliability

The results showed that the total questionnaire’s Spearman-Brown split-half reliability coefficient was 0.925, and the Spearman-Brown split-half reliability coefficients for each dimension ranged from 0.739 to 0.956, indicating good internal consistency of the questionnaire.

#### Test-Retest Reliability

In this study, test-retest reliability was assessed by readministering the questionnaire to 20 individuals 2 weeks after the initial administration. The results showed that the total questionnaire’s test-retest reliability coefficient was 0.925, while the coefficients for each dimension ranged from 0.875 to 0.933, all exceeding 0.700. These findings indicate that the questionnaire exhibits good stability over time. See Table 5 for detailed results.

**Table 5:** Reliability analysis of the digital health literacy questionnaire in older adults.

Dimensions	Item numbers	Cronbach α coefficient	Split-half reliability	Test-retest reliability
Information	12	0.950	0.946	0.903
Behavior	8	0.952	0.956	0.933
Safety	5	0.916	0.837	0.897
Interaction	7	0.939	0.888	0.880
Content	4	0.880	0.888	0.891
Attitude	3	0.819	0.739	0.875
Digital health literacy	39	0.976	0.925	0.925

## Discussion

### DIGCOMP-Based Framework for Assessing Digital Health Literacy

The study utilized the DIGCOMP framework as a conceptual foundation, integrating expert opinions and empirical research to refine the structure and content of the questionnaire. The final instrument assesses 6 dimensions of DHL and demonstrates strong psychometric properties. This provides a solid empirical basis to support health professionals in developing individualized intervention strategies and optimizing health service delivery.

### Questionnaire Formation Is Meaningful

As the global population ages, older adults are becoming a priority in the health care sector [30]. With the rapid advancement of digital technology, they face multiple challenges in managing their health. Cognitive decline and limited digital training can make it difficult for them to use digital tools for health management. This can hinder their access to essential health information and resources, as well as services such as online appointments, telemedicine consultations, and electronic health records. As a result, these barriers may increase the risk of medication errors and exacerbate health inequalities.

From the perspective of enhancing older adults’ engagement in health management, improving DHL is a top priority in developing a digital service system for older adults [11]. The concept of DHL is continuously evolving and requires distinct competencies compared with eHL. Although both are related, they differ significantly in their practical application for older adults. eHL primarily focuses on their ability to process and apply health information online, such as searching for disease-related information, comparing data from multiple authoritative sources, and identifying false medical advertisements. It emphasizes critical thinking and the ability to assess the authenticity of health information [14,31]. By contrast, DHL emphasizes older adults’ ability to effectively use digital health devices, such as smart blood pressure monitors or health tracking bracelets, to manage their health data. It involves applying digital tools to adjust exercise routines, modify daily diets based on professional advice, and engage in self-care. This requires a certain level of technical competence. Additionally, DHL highlights the importance of exchanging and evaluating health information, as well as ensuring privacy protection in digital interactions [3]. Therefore, if an assessment tool is not designed based on the latest DHL concepts, it may fail to align with the essential skills older adults need to develop and demonstrate. There will be no direct comparison between the results of different studies, which may limit their reproducibility and generalizability. However, most existing assessment tools for older adults have been developed based on eHL, without considering the unique characteristics of this population [12-16]. Currently, there is no specific DHL assessment tool designed for older adults. The questionnaire developed in this study addresses this gap by evaluating the current status of DHL in older adults and identifying areas where digital health competencies need improvement. This provides a foundation for developing appropriate intervention strategies and targeted health education, making it highly relevant in the current digital health landscape.

### Questionnaire Construction Process Is Reasonable

This study conducted a comprehensive review of relevant literature and government policy documents. Using the widely accepted DHL concept, the DIGCOMP framework was adopted as the theoretical foundation to ensure the questionnaire’s scientific rigor. The initial framework was developed through face-to-face semistructured interviews with 6 experts and 5 rounds of research group discussions. To ensure content validity, 3 rounds of expert consultations were conducted with 16 specialists from hospitals, academic institutions, and technology companies. These experts, with extensive experience in geriatric care, provided valuable insights into the characteristics and needs of older adults. The recommendations provided by these experts were comprehensive and highly instructive. The questionnaire development process emphasized aspects that had been overlooked in previous assessment tools, including interaction (both with others and with devices), content editing (such as information integration and copyright awareness), and security (covering privacy protection, device security, and health-related safety). The questionnaire items were designed to align with the realities of older adults’ daily lives and health care experiences, focusing on potential challenges they may face when using digital technologies to access, process, communicate, and understand health information and services. To ensure its relevance and usability, a presurvey was conducted with 10 older adults from diverse backgrounds. Through individual interviews, participants’ experiences with completing the questionnaire were observed and recorded. Based on these insights, the questionnaire was simplified in terms of language and optimized in layout to enhance its applicability and improve data collection efficiency. Additionally, complex terminology was replaced with more accessible wording to make it easier for older adults to respond. For essential terms that needed to be retained but might be difficult to understand, clear explanations were added to ensure clarity.

### The Contents of the Questionnaire Are Scientific

The study refined the questionnaire entries through empirical research. The research team conducted multiple discussions on the professional significance of each entry, making modifications and deletions based on predefined criteria and expert knowledge.

The “Information” dimension evaluates older adults’ ability to access, assess, and utilize health information through digital devices, as well as their understanding of digital health. This dimension is a core component of DHL measurement. In our study, 12 of the original 14 items in this dimension were retained in the final questionnaire. The item “I will check if the health information is of value to me” was removed after discussions with the research team, as it was semantically similar to other items in the same dimension and had a factor loading below 0.4. Additionally, the item “I care about health information related to myself” was reassigned to the “Attitude” dimension.

The “Behavior” dimension assesses older adults’ ability to manage their health using digital devices and their understanding of how digital technology can support their well-being [32]. During the revision process, the item “I think digital health information is trustworthy,” originally part of the “Attitude” dimension, was removed, while the remaining items were reassigned to the “Behavior” dimension. Additionally, through expert consultations and group discussions, the “Problem-Solving” dimension from the DIGCOMP framework was subdivided into the “Attitude” and “Behavior” dimensions. As a result, the empirical study justified the reclassification of certain items between these 2 dimensions.

The “Safety” dimension assesses older adults’ ability to protect their personal information and physical and mental well-being while managing their health through digital devices. This competency, which has not been explicitly addressed in the concept of eHL [3], is crucial for ensuring the safe and effective participation of older adults in digital health activities [17,19]. During the questionnaire evaluation, most items remained within this dimension. However, the item “I believe that personal health data can be accessed by medical staff directly involved in the treatment” was removed due to insufficient factor loading. Additionally, the item “I know how to protect personal digital devices from cyber attacks” exhibited double loading with similar values. Apart from these adjustments, no attributional changes were made to the remaining items.

The “Interaction” dimension in evaluating older adults’ DHL emphasizes their ability to communicate health information with individuals, health care platforms, artificial intelligence, and other digital systems. Previous research has primarily focused on older adults’ ability to communicate with people in the context of DHL [33], likely due to the traditional perspective that health information exchange occurs mainly between individuals. However, with advancements in technology, the ability of older adults to effectively interact with digital devices is not only crucial for accessing timely and accurate health information but also directly influences the quality of their health management and self-care. During the evaluation of questionnaire entries, 1 item exhibited multiple loadings, and 3 items were reassigned to other dimensions after discussion. No items from other dimensions were added to this category.

The “Content” dimension emphasizes older adults’ ability to integrate and enhance digital health information. While it is related to other dimensions, it is distinct in its focus on evaluating overall competencies [17]. In this dimension, we incorporated the item, “I have participated in online health knowledge seminars, health care experience-sharing activities, and similar events in the past 12 months,” which was originally part of the “Interaction” dimension. Although each dimension of the DIGCOMP framework has unique characteristics, there are inherent overlaps and interconnections between them [17]. The “Content creation” competency in the DIGCOMP framework includes “Sharing information and content,” which aligns with the competencies assessed in the “Content” dimension. As interaction serves as a prerequisite for content creation and addressing health concerns [17], this reallocation is justified.

Additionally, we identified 2 entries from the original “Interaction” dimension—“I can adhere to the correct political direction in my online behavior” and “I can refrain from fabricating or spreading false and unverified health information”—as well as 1 entry from the former “Information” dimension—“I care about health information related to myself.” These 3 entries differ significantly in connotation from the other items in their respective dimensions. After an in-depth group discussion, we consolidated them into a newly established “Attitude” dimension. This dimension was created to assess older adults’ sense of responsibility, political sensitivity, and concern for personal health information when managing digital health information.

### The Implications of This Study for Future Research

The DHL questionnaire for older adults developed in our study provides future researchers with a standardized measurement tool, facilitating comparative studies across different regions and populations. Our findings also underscore the importance of enhancing DHL in older adults. Future research can build on our study to further refine the questionnaire’s structure and content. Additionally, researchers can explore targeted educational interventions in greater detail, such as developing digital skills training programs tailored for older adults and designing user-friendly digital health education resources. Additionally, researchers can use these findings to develop more targeted policies, such as promoting digital inclusion initiatives for older adults and establishing a comprehensive community support system. This could include community health education programs, volunteer service networks, and mutual aid groups. Such measures will effectively enhance the DHL of older adults, ultimately improving their overall health and quality of life and supporting the goal of healthy aging.

### Limitations

There are some limitations to our study. The questionnaire developed was a self-assessment tool, which may lack objectivity. Older adults completing self-assessment questionnaires might select responses they believe are more socially acceptable or aligned with the investigators’ expectations rather than their true thoughts, feelings, or behaviors regarding digital health. This tendency could introduce social desirability and response biases, potentially reducing data accuracy. Additionally, self-assessments may be affected by recall bias, as older adults might struggle with memory inaccuracies or overestimate their DHL, leading to discrepancies between their reported and actual abilities. Methods such as scenario modeling could be considered for objective evaluation in the future. Additionally, our study used a web-based survey, requiring older adults to complete the questionnaire independently, which may have excluded those with poorer numerical ability and health. These individuals may prefer obtaining health information through traditional channels (such as television, newspapers, and face-to-face communication), have lower familiarity with and less frequent use of the internet and digital technologies, and may face difficulties in accessing and interpreting health information. However, excluding them may result in health needs and issues specific to certain groups of older adults being inadequately explored, potentially affecting the ubiquity and applicability of the findings. Future research could incorporate both online and traditional face-to-face surveys. Additionally, a more concise and comprehensible questionnaire should be designed, and older adults should be provided with the necessary digital skills training to facilitate their participation in research. Furthermore, this questionnaire has only undergone psychometric testing among Chinese older adults. Therefore, cross-linguistic validation is needed to confirm its cultural invariance.

### Conclusions

As scientific and technological advancements intertwine with theoretical research developments, the concept of DHL must be continuously explored and updated. Our study developed a DHL questionnaire for older adults based on the latest DHL concepts, assessing 6 dimensions: Information, Behavior, Security, Interaction, Content, and Attitude. The questionnaire closely reflects the real-life experiences of older adults and demonstrates strong differentiation, reliability, and practicality. It provides health-related practitioners with a scientifically sound and feasible tool for accurately and comprehensively assessing the current status of DHL in older adults. Future research should conduct an in-depth assessment of the questionnaire’s applicability across different linguistic and cultural contexts and evaluate its validity among older adults with varying health statuses, educational backgrounds, and socioeconomic conditions. Additionally, the effectiveness of the questionnaire in specific intervention programs should be assessed to validate its practical guiding role. Furthermore, it is essential to explore ways to integrate assessment results into targeted health promotion strategies and incorporate theories and methods from multiple disciplines, such as psychology, sociology, and education. Through interdisciplinary collaboration, DHL enhancement initiatives for older adults can be more effectively implemented, providing comprehensive support for their health management.

## Appendix

Multimedia Appendix 1 The implementation and analysis processes of the semistructured interviews.

Multimedia Appendix 2 Digital Health Literacy Survey for Older Adults.

Multimedia Appendix 3 Item analysis.

Multimedia Appendix 4 Exploratory factor analysis factor loadings matrix of the digital health literacy (DHL) Questionnaire for older adults.

Multimedia Appendix 5 Criterion-related validity of the digital health literacy (DHL) Questionnaire in older adults.

## Abbreviations

AVE: average variance extracted
DHL: digital health literacy
DIGCOMP: Digital Competence Framework
EFA: exploratory factor analysis
eHEALS: eHealth Literacy Scale
eHL: eHealth literacy
HL: health literacy
I-CVI: item-level content validity index
KMO: Kaiser-Meyer-Olkin
S-CVI/Ave: scale-level content validity index

## Data Availability

The data sets generated or analyzed during this study are available from the corresponding author (WL) on reasonable request.
