# Supplementary material for: Digital Health Literacy Questionnaire for Older Adults: Instrument Development and Validation Study
Source: J Med Internet Res. 2025 Mar 19;27:e64193. doi: 10.2196/64193 (PMC11966078; doi:10.2196/64193)
Supplement: Multimedia Appendix 1 [file jmir_v27i1e64193_app1.docx]

**The implementation and analysis processes of the semi-structured interviews**

**Semi-structured interviews**

The 6 experts were interviewed face-to-face by interviewers who had received standardized training in qualitative research methods. The outline of the interviews included the following questions:

(1) What do you think should be included in the evaluation index of DHL for older adults?

(2) What do you think should be modified?

The interviews were conducted in a quiet, comfortable hospital conference room, with each session lasting 30 to 45 minutes. The interviewer actively listened and followed up on the participants' responses when appropriate, carefully noting non-verbal cues such as facial expressions, body language, and speaking pace. A total of 220 minutes of semi-structured interviews were conducted. The results indicated that the experts found DIGCOMP 2.0 to be an appropriate foundational theoretical framework, though modifications were necessary. Consequently, 5 rounds of research group discussionss were held to continuously update the questionnaire and to align make it better with China's national context and cultural background.

**Modifications were made in the research group discussionss as follows:**

**Factors**

1. Based on DIGCOMP 2.0, the primary factors should include information, communication, content, safety, and problem solving. One expert suggested adding 'practical operation' as an additional factor, considering its relevance for older adults. The group agreed and included this factor to enhance the questionnaire's practicality.
2. An expert proposed renaming the factors 'Problem Solving' and 'Practical Operation' to 'Attitude' and 'Behavior,' respectively. While the original factors aimed to assess older adults' situation with digital health devices, they were seen as semantically distant from the other four factors. Renaming these as 'Attitude' and 'Behavior' allows a clearer distinction between application-focused and attitude-focused aspects, creating more coherence within the questionnaire.
3. An expert noted redundancy between the factors 'Information' and 'Content' as well as between 'Communication' and 'Practical Operation'. They suggested renaming 'Communication' to 'Interaction'. 'Communication' refers to the exchange of information, where individuals provide what they have to others. In contrast, 'Interaction' encompasses not only the exchange of information but also the generation of new ideas, thoughts, and needs during the process of communication. This term has a broader semantic scope, better capturing the concept of digital health literacy, where individuals continuously acquire, process, share, and understand health information and services to make informed health decisions. Such decisions ultimately support and improve both individual and collective health. Therefore, the group adopted this suggestion.
4. One expert suggested adding 'economic capability and educational level' as factors, while another recommended including a 'follow-up: understanding subsequent health status' factor. However, after discussion, the research group thought that economic capability, educational level, and health status are more appropriate for inclusion in the demographic survey and will be added in the subsequent questionnaire stage. The final primary factors were established as Information, Behavior, Safety, Interaction, Content, and Attitude.

**Items**

1. The original items included 'Checking the publisher's professional background and user or reader evaluations' and 'Verifying the information source and understanding the channels through which information is obtained.' Three experts identified semantic redundancy between these two items. Consequently, the two items were merged into the new Item: '1: I pay attention to whether health information is released and disseminated by official or authoritative institutions.'
2. The original item 'I know how to publish, repost, or share health information on online platforms' was revised for more specificity, suggested by two experts. It has been modified to: 'I use information dissemination platforms (such as Weibo, WeChat Moments, etc.) to share information.'
3. An expert expressed concerns that the original item 'Understanding the impact of digital technology on one's environment' was ambiguous and overly broad. The influence of digital technology on the environment encompasses various aspects, including physical and social environments, which may be difficult for many older adults to grasp, leading to potential misunderstandings. Additionally, this item primarily aimed to assess older adults' awareness of the negative effects of the internet, including potential physical, psychological, and financial risks. Thus, the research group adopted the expert's suggestion and revised the item to: '25. I know the potential security risks in the online environment.'
