# Supplementary material for: Digital Health Literacy Questionnaire for Older Adults: Instrument Development and Validation Study
Source: J Med Internet Res. 2025 Mar 19;27:e64193. doi: 10.2196/64193 (PMC11966078; doi:10.2196/64193)
Supplement: Multimedia Appendix 2 [file jmir_v27i1e64193_app2.docx]

**老年人数字健康素养调查问卷**

**第一部分 知情同意书**

尊敬的先生/女士：

您好！

我们是“老年人数字健康素养问卷开发”课题组的调查人员，正在开展一项关于老年人数字健康的调查研究，我们将通过了解您数字健康素养现状，以期为老年人数字健康素养问卷开发相关研究提供参考。本次调查内容包括一般情况调查和量表填写两大部分，您只需选择您认可的答案，问卷填写大约需要10分钟左右的时间。

我们会对您提供的个人资料严格保密，而且只有本课题组成员才能接触这些资料，课题结束后我们会将资料进行封存，敬请放心。您有权拒绝本次调查，或在调查中的任何时候选择退出，本次调查完全遵守自愿原则，不会影响您与您亲朋好友的生活。

真诚地希望您能同意参加本次调查，您所提供的资料对我们今后的研究十分重要，感谢您的参与！

调查人员已向我解释了本次调查的目的和注意事项，并且我已了解本次调查的目的和注意事项。我同意并自愿参与本次调查。

参与者签名：

签名日期： 年 月 日

**第二部分 一般资料调查问卷**

指导语：请您仔细阅读题目及选项，并根据实际情况在对应的选项划“√”或在横线处补充。

1. **性别：**□男 □女
2. **年龄：**  岁
3. **文化程度：**□初中及以下 □高中或中专 □大专或大学 □研究生
4. **婚姻状况：**□未婚 □已婚 □离异 □丧偶
5. **居住情况：**□独居 □与配偶居住 □与子女居住 □与配偶及子女居住 □其他
6. **家庭人均月收入（元）：**

□2000以下 □2000-4000 □4000-6000 □6000-8000 □8000-10000 □10000以上

1. **经济来源：**□退休金 □子女赡养 □再就业收入 □低保 □其他
2. **医疗付费方式：**

□城镇医保 □农村医保 □职工医保 □商业医疗保险 □自费

1. **慢性病患病情况：**□无 □1种 □2种 □3种及3种以上

**第三部分 电子健康素养量表**

指导语：请您仔细阅读题目及选项，并根据实际情况在对应的选项划“√”或在横线处补充。

| **条目** | **非常不符合** | **不符合** | **不确定** | **符合** | **非常符合** |
| --- | --- | --- | --- | --- | --- |
| 1.我知道从网络上可以获取的卫生信息有哪些 |  |  |  |  |  |
| 2.我知道从网络上哪里可以获取有用的卫生资源信息 |  |  |  |  |  |
| 3.我知道如何上网查找有用的卫生资源信息 |  |  |  |  |  |
| 4.我知道如何利用网络来解答自己的健康问题 |  |  |  |  |  |
| 5.我知道如何利用获取的网络卫生资源信息帮助自己 |  |  |  |  |  |
| 6.我具备评价网络卫生资源信息好坏的技能 |  |  |  |  |  |
| 7.我能够区分网络上高质量和低质量的卫生资源信息 |  |  |  |  |  |
| 8.我对应用网络信息做出健康相关决定充满自信 |  |  |  |  |  |

**第四部分 老年人数字健康素养问卷**

指导语：请您仔细阅读题目及选项，并根据实际情况在对应的选项划“√”。

| **条目** | **非常不符合** | **不符合** | **不确定** | **符合** | **非常符合** |
| --- | --- | --- | --- | --- | --- |
| **一、信息** |  |  |  |  |  |
| 1.我了解数字健康技术（如可穿戴设备、智能健康电子产品等） |  |  |  |  |  |
| 2.我了解数字技术可用于健康管理或健康促进（如健康医疗移动应用服务等） |  |  |  |  |  |
| 3.我关心与自己有关的健康信息 |  |  |  |  |  |
| 4.我可以通过数字设备或软件浏览、检索、获取健康信息 |  |  |  |  |  |
| 5.我会关注健康信息更新 |  |  |  |  |  |
| 6.我使用过数字健康设备或软件记录个人健康信息 |  |  |  |  |  |
| 7.我了解数字健康设备或软件可用于存储个人健康信息 |  |  |  |  |  |
| 8.我关注健康信息是否为官方或权威机构发布和传播 |  |  |  |  |  |
| 9.我会检查健康信息对自己是否有价值 |  |  |  |  |  |
| 10.我会将同类健康信息进行比较 |  |  |  |  |  |
| 11.我收到健康信息后不会立刻分享给其他人，而是会打开看看内容再说 |  |  |  |  |  |
| 12.我对获取的健康信息始终保持警惕性，不会轻信 |  |  |  |  |  |
| 13.我会从其他渠道印证健康信息是否正确 |  |  |  |  |  |
| 1. 我可以判断健康信息是否与商业利益相关（例如含有产品广告） |  |  |  |  |  |
| **二、交互** |  |  |  |  |  |
| 1.我熟悉数字设备或软件使用界面 |  |  |  |  |  |
| 2.我可以用数字设备或软件与他人交流健康信息 |  |  |  |  |  |
| 3.我可以用数字设备或软件与人工智能（AI）交流健康信息 |  |  |  |  |  |
| 4.我可以与他人在互联网上分享信息 |  |  |  |  |  |
| 5.我可以与他人线下分享自己在线上获取的信息 |  |  |  |  |  |
| 6.我会使用信息发布平台（如微博、微信朋友圈等）分享信息 |  |  |  |  |  |
|  | **非常不符合** | **不符合** | **不确定** | **符合** | **非常符合** |
| 7.我会效仿健康信息中提到的健康促进行为或健康管理方法 |  |  |  |  |  |
| 8.我会向专业人员求证自己获取的信息是否真实有效 |  |  |  |  |  |
| 9.我在过去12个月内曾参与线上健康知识讲座、健康保健经验交流分享活动等 |  |  |  |  |  |
| 10.我能做到不编造、传播虚假的、未经证实的健康信息 |  |  |  |  |  |
| 11.我能做到在网络行为中坚持正确的政治方向 |  |  |  |  |  |
| **三、内容** |  |  |  |  |  |
| 1.我可以编辑和改进自己或他人创建的健康内容 |  |  |  |  |  |
| 2.我可以将多渠道健康信息整合并重新阐述 |  |  |  |  |  |
| 3.我能做到保护原创作品完整性，引用标注信息来源 |  |  |  |  |  |
| **四、安全** |  |  |  |  |  |
| 1.我知道如何保护个人数字设备免受网络攻击 |  |  |  |  |  |
| 2.我不会点击不安全的网络链接；不会进入提醒存在风险的网站 |  |  |  |  |  |
| 3.我认为自己有个人数据的所有权，只有经过自己授权，他人才能获取自己的个人健康数据信息 |  |  |  |  |  |
| 4.我认为个人健康数据可被直接参与诊疗的医务人员获取 |  |  |  |  |  |
| 5.我认为对于未经授权的数据获取或保存不当引起的数据泄露，自己有权追究法律责任 |  |  |  |  |  |
| 6.我可以避免与使用数字技术有关的、威胁身心健康的健康风险 |  |  |  |  |  |
| 1. 我知道网络环境可能存在的不安全隐患 |  |  |  |  |  |
| **五、态度** |  |  |  |  |  |
| 1.我习惯使用数字服务处理健康信息 |  |  |  |  |  |
| 2.如有需要的话，我认为我能坚持使用数字健康工具 |  |  |  |  |  |
| 3.我认为数字健康信息可信 |  |  |  |  |  |
| 4.我认为数字技术的使用有利于自身的健康管理 |  |  |  |  |  |
| **六、行为** |  |  |  |  |  |
| 1.我知道什么时候、怎样使用以及使用什么健康信息 |  |  |  |  |  |
| 2.我知道应选择哪种数字健康工具满足健康需求 |  |  |  |  |  |
|  | **非常不符合** | **不符合** | **不确定** | **符合** | **非常符合** |
| 1. 我可以判断数字健康工具是否可信 |  |  |  |  |  |
| 4.在使用数字健康工具过程中，我能根据实际情况调整自己的使  用频率、强度、方式 |  |  |  |  |  |
| 5.我能够使用数字设备或电子健康产品或软件 |  |  |  |  |  |
| 6.我知道如何利用数字健康工具上的记录为我的日常健康管理提供参考 |  |  |  |  |  |
| 7.我知道如何利用数字健康工具对我的健康行为进行追踪 |  |  |  |  |  |

**Digital Health Literacy Questionnaire for Older Adults**

**Part 1 Informed Consent Form**

Dear Sir/Madam:

We are the investigators of the ‘Development of Digital Health Literacy Questionnaire for the Older Adults’ project team, and we are conducting a survey on the digital health of the older adults. We will investigate the current status of your digital health literacy, in order to provide reference for the development of the Digital Health Literacy Questionnaire for the Older Adults. The survey consists of two major parts: general survey and questionnaire filling, you only need to choose the answers you agree with, and it takes about 10 minutes to fill in the questionnaire.

Please be assured that the personal information you provide will be kept strictly confidential, and only members of this team will have access to this information, which will be sealed at the end of the project. You have the right to refuse this survey, or opt out at any time during the survey. This survey is completely voluntary and will not affect your life with your friends and family.

We sincerely hope that you will agree to participate in this survey, the information you provide is very important to our future research, thank you for your participation!

Kind regards!

The purpose and considerations of this survey have been explained to me by the investigator and I have understood the purpose and considerations of this survey. I agree and voluntarily participate in this survey.

Sign：

Date:

**Part 2 General information questionnaire**

Guideline: Please read the questions and options carefully and tick the corresponding option or add in the crosses as appropriate.

1. **Gender**：□Male □Female
2. **Age**：
3. **Educational level**：□Junior high and below □High school/secondary vocational □College/university □Postgraduates
4. **Marital status**：□Unmarried □Married □Divorce □Widow
5. **Residential situation**：□Living alone □Living with spouse □Living with children □Living with spouse and children □Other
6. **Household monthly income**：

□<2000 CNY □2000-4000CNY □4000-6000CNY □6000-8000CNY □8000-10000CNY □1> 10000 CNY

1. **Primary source of income**：□Pension □Child support□Re-employment income □Subsistence allowance □Other
2. **Medical Payment Methods**：

□Urban Medical Insurance □Rural Medical Insurance □Employee Medical Insurance □Commercial Medical Insurance □Self-financed

1. **Chronic Disease**：□Without □1 □2 □≥3

**Part 3 The eHealth Literacy Scale (eHEALS)**

Guideline: Please read the questions and options carefully and tick the corresponding option or add in the crosses as appropriate.

| **Items** | **Strongly disagree** | **Disagree** | **Uncertain** | **Agree** | **Strongly agree** |
| --- | --- | --- | --- | --- | --- |
| 1. I know how to find helpful health resources on the Internet |  |  |  |  |  |
| 2. I know how to use the Internet to answer my health questions |  |  |  |  |  |
| 3. I know what health resources are available on the Internet |  |  |  |  |  |
| 4. I know where to find helpful health resources on the Internet |  |  |  |  |  |
| 5. I know how to use the health information I find on the Internet to help me |  |  |  |  |  |
| 6. I have the skills I need to evaluate the health resources I find on the Internet |  |  |  |  |  |
| 7. I can tell high quality from low quality health resources on the Internet |  |  |  |  |  |
| 8. I feel confident in using information from the Internet to make health decisions |  |  |  |  |  |

**Part 3 Digital Health Literacy Questionnaire for Older Adults**

Guideline: Please read the questions and options carefully and tick the corresponding option or add in the crosses as appropriate.

| **Items** | **Strongly disagree** | **Disagree** | **Uncertain** | **Agree** | **Strongly agree** |
| --- | --- | --- | --- | --- | --- |
| **Information** |  |  |  |  |  |
| 1. I understand digital health technologies (such as wearable devices, smart health electronic products, etc.). |  |  |  |  |  |
| 2. I understand that digital technologies can be used for health management or health promotion (such as health and medical mobile applications). |  |  |  |  |  |
| 3. I care about health information related to myself. |  |  |  |  |  |
| 4. I can browse, search, and obtain health information through digital devices or software. |  |  |  |  |  |
| 5. I pay attention to updates on health information |  |  |  |  |  |
| 6. I have used digital health devices or software to record personal health information. |  |  |  |  |  |
| 7. I understand that digital health devices or software can be used to store personal health information. |  |  |  |  |  |
| 1. I pay attention to whether health information is released and disseminated by official or authoritative institutions. |  |  |  |  |  |
| 9. I will check if the health information is of value to me. |  |  |  |  |  |
| 10. I compare similar health information. |  |  |  |  |  |
| 11. I do not immediately share health information with others after receiving it but first check the content. |  |  |  |  |  |
|  | **Strongly disagree** | **Disagree** | **Uncertain** | **Agree** | **Strongly agree** |
| 1. I remain vigilant about the health information I obtain and do not easily believe it. |  |  |  |  |  |
| 1. I verify the correctness of health information from other sources. |  |  |  |  |  |
| 14. I can judge whether health information is related to commercial interests (e.g., contains product advertisements) |  |  |  |  |  |
| **Interaction** |  |  |  |  |  |
| 15. I am familiar with the user interface of digital devices or software. |  |  |  |  |  |
| 16. I can use digital devices or software to communicate health information with others. |  |  |  |  |  |
| 17. I can use digital devices or software to communicate health information with artificial intelligence (AI). |  |  |  |  |  |
| 18. I can share information with others on the internet. |  |  |  |  |  |
| 19. I can share information I obtained online with others offline. |  |  |  |  |  |
| 20. I use information dissemination platforms (such as Weibo, WeChat Moments, etc.) to share information. |  |  |  |  |  |
| 21. I imitate the health-promoting behaviors or health management methods mentioned in health information. |  |  |  |  |  |
| 22. I would seek confirmation from a professional that the information I have obtained is true and valid. |  |  |  |  |  |
| 23. In the past 12 months, I have participated in online health lectures and health care experience sharing activities. |  |  |  |  |  |
|  | **Strongly disagree** | **Disagree** | **Uncertain** | **Agree** | **Strongly agree** |
| 24. I do not fabricate or spread false, unverified health information. |  |  |  |  |  |
| 25. I adhere to correct political direction in online behavior. |  |  |  |  |  |
| **Content** |  |  |  |  |  |
| 26. I can edit and improve health content created by myself or others. |  |  |  |  |  |
| 27. I can integrate health information from multiple sources and rephrase it. |  |  |  |  |  |
| 28. I can protect the integrity of original works and cite sources when referencing. |  |  |  |  |  |
| **Safety** |  |  |  |  |  |
| 29. I know how to protect personal digital devices from cyber attacks. |  |  |  |  |  |
| 30. I do not click on unsafe web links; I do not visit websites that are flagged as risky. |  |  |  |  |  |
| 31. I believe I have ownership of personal data, and others can only obtain my personal health data with my authorization. |  |  |  |  |  |
| 32. I believe that personal health data can be accessed by medical staff directly involved in the treatment. |  |  |  |  |  |
| 33. I believe I have the right to pursue legal responsibility for unauthorized data acquisition or improper data storage that leads to data breaches. |  |  |  |  |  |
| 34. I can avoid health risks related to the use of digital technologies that threaten physical and mental health. |  |  |  |  |  |
| 35. I know the potential security risks in the online environment. |  |  |  |  |  |
|  | **Strongly disagree** | **Disagree** | **Uncertain** | **Agree** | **Strongly agree** |
| **Attitude** |  |  |  |  |  |
| 36. I am used to using digital services to handle health information. |  |  |  |  |  |
| 37. If necessary, I think I can persist in using digital health tools. |  |  |  |  |  |
| 38. I think digital health information is credible. |  |  |  |  |  |
| 39. I believe the use of digital technologies is beneficial for my health management. |  |  |  |  |  |
| **Behavior** |  |  |  |  |  |
| 40. I know when, how, and what health information to use. |  |  |  |  |  |
| 41. I know which digital health tools to choose for my health needs. |  |  |  |  |  |
| 42. I can judge whether digital health tools are trustworthy. |  |  |  |  |  |
| 43. During the use of digital health tools, I can adjust my frequency, intensity, and methods based on the actual situation. |  |  |  |  |  |
| 44. I can use digital devices or electronic health products or software. |  |  |  |  |  |
| 45. I know how to use the records on digital health tools to inform my daily health management. |  |  |  |  |  |
| 46. I know how to use digital health tools to track my health behavior . |  |  |  |  |  |

**Note: This English version is a direct translation from the author and has not been culturally adjusted.**
