# Supplementary material for: Digital Health Literacy Questionnaire for Older Adults: Instrument Development and Validation Study
Source: J Med Internet Res. 2025 Mar 19;27:e64193. doi: 10.2196/64193 (PMC11966078; doi:10.2196/64193)
Supplement: Multimedia Appendix 3 [file jmir_v27i1e64193_app3.docx]

**Multimedia Appendix 3:** Item Analysis

| Items | Critical ratio method | Correlation coefficient between the items and the total score | | | Homogeneity test | | Items failed to meet criteria | Results |
| --- | --- | --- | --- | --- | --- | --- | --- | --- |
|  | Critical value | Items | Corrected | Cronbach's α after deleting any item | Communality | Factor loading |  |  |
| Item 1 | 11.472^c^ | 0.633 ^b^ | 0.613 | 0.963 | 0.568 | 0.697 | 0 | Remain |
| Item 2 | 11.823^c^ | 0.596 ^b^ | 0.574 | 0.963 | 0.556 | 0.494 | 0 | Remain |
| Item 3 | 9.241^c^ | 0.514 ^b^ | 0.488 | 0.964 | 0.469 | 0.441 | 0 | Remain |
| Item 4 | 11.490^c^ | 0.646 ^b^ | 0.625 | 0.963 | 0.529 | 0.478 | 0 | Remain |
| Item 5 | 11.838^c^ | 0.639 ^b^ | 0.618 | 0.963 | 0.546 | 0.507 | 0 | Remain |
| Item 6 | 11.916^c^ | 0.641 ^b^ | 0.618 | 0.963 | 0.483 | 0.603 | 0 | Remain |
| Item 7 | 12.382^c^ | 0.667 ^b^ | 0.647 | 0.963 | 0.546 | 0.455 | 0 | Remain |
| Item 8 | 11.936^c^ | 0.546 ^b^ | 0.522 | 0.964 | 0.479 | 0.406 | 0 | Remain |
| Item 9 | 12.527^c^ | 0.577 ^b^ | 0.553 | 0.963 | 0.537 | 0.428 | 0 | Remain |
| Item 10 | 10.777^c^ | 0.534 ^b^ | 0.511 | 0.964 | 0.622 | 0.646 | 0 | Remain |
| Item 11 | 10.768^c^ | 0.543 ^b^ | 0.518 | 0.964 | 0.534 | 0.539 | 0 | Remain |
| Item 12 | 9.889^c^ | 0.506 ^b^ | 0.479 | 0.964 | 0.627 | 0.688 | 0 | Remain |
| Item 13 | 12.172^c^ | 0.567 ^b^ | 0.544 | 0.963 | 0.577 | 0.627 | 0 | Remain |
| Item 14 | 11.933^c^ | 0.591 ^b^ | 0.569 | 0.963 | 0.562 | 0.497 | 0 | Remain |
| Item 15 | 13.201^c^ | 0.700 ^b^ | 0.681 | 0.963 | 0.616 | 0.581 | 0 | Remain |
| Item 16 | 11.905^c^ | 0.664 ^b^ | 0.643 | 0.963 | 0.548 | 0.500 | 0 | Remain |
| Item 17 | 12.130^c^ | 0.663 ^b^ | 0.640 | 0.963 | 0.614 | 0.640 | 0 | Remain |
| Item 18 | 12.125^c^ | 0.663 ^b^ | 0.641 | 0.963 | 0.561 | 0.567 | 0 | Remain |
| Item 19 | 11.215^c^ | 0.637 ^b^ | 0.615 | 0.963 | 0.506 | 0.436 | 0 | Remain |
| Item 20 | 11.118^c^ | 0.630 ^b^ | 0.606 | 0.963 | 0.529 | 0.586 | 0 | Remain |
| Item 21 | 11.389^c^ | 0.650 ^b^ | 0.628 | 0.963 | 0.547 | 0.501 | 0 | Remain |
| Item 22 | 13.212^c^ | 0.666 ^b^ | 0.646 | 0.963 | 0.617 | 0.581 | 0 | Remain |
| Item 23 | 12.043^c^ | 0.614 ^b^ | 0.587 | 0.963 | 0.543 | 0.375 | 0 | Remain |
| Item 24 | 9.315^c^ | 0.484 ^b^ | 0.460 | 0.964 | 0.461 | 0.508 | 0 | Remain |
| Item 25 | 9.441^c^ | 0.529 ^b^ | 0.506 | 0.964 | 0.612 | 0.469 | 0 | Remain |
| Item 26 | 11.203^c^ | 0.587 ^b^ | 0.560 | 0.963 | 0.627 | 0.558 | 0 | Remain |
| Item 27 | 12.580^c^ | 0.642 ^b^ | 0.619 | 0.963 | 0.562 | 0.390 | 1 | Remain |
| Item 28 | 11.765^c^ | 0.660 ^b^ | 0.638 | 0.963 | 0.599 | 0.590 | 0 | Remain |
| Item 29 | 10.681^c^ | 0.587 ^b^ | 0.564 | 0.963 | 0.516 | 0.517 | 0 | Remain |
| Item 30 | 9.933^c^ | 0.544 ^b^ | 0.521 | 0.964 | 0.618 | 0.672 | 0 | Remain |
| Item 31 | 10.032^c^ | 0.551 ^b^ | 0.527 | 0.964 | 0.497 | 0.365 | 1 | Remain |
| Item 32 | 10.186^c^ | 0.522 ^b^ | 0.494 | 0.964 | 0.553 | 0.541 | 0 | Remain |
| Item 33 | 9.933^c^ | 0.537 ^b^ | 0.514 | 0.964 | 0.524 | 0.627 | 0 | Remain |
| Item 34 | 12.171^c^ | 0.656 ^b^ | 0.636 | 0.963 | 0.516 | 0.443 | 0 | Remain |
| Item 35 | 8.647^c^ | 0.468 ^b^ | 0.442 | 0.964 | 0.446 | 0.526 | 0 | Remain |
| Item 36 | 14.494^c^ | 0.744 ^b^ | 0.725 | 0.963 | 0.667 | 0.672 | 0 | Remain |
| Item 37 | 13.413^c^ | 0.739 ^b^ | 0.721 | 0.963 | 0.677 | 0.602 | 0 | Remain |
| Item 38 | 13.652^c^ | 0.705 ^b^ | 0.685 | 0.963 | 0.599 | 0.612 | 0 | Remain |
| Item 39 | 13.345^c^ | 0.717 ^b^ | 0.699 | 0.963 | 0.653 | 0.555 | 0 | Remain |
| Item 40 | 12.026^c^ | 0.644 ^b^ | 0.625 | 0.963 | 0.500 | 0.485 | 0 | Remain |
| Item 41 | 12.083^c^ | 0.635 ^b^ | 0.615 | 0.963 | 0.617 | 0.526 | 0 | Remain |
| Item 42 | 13.227^c^ | 0.667 ^b^ | 0.647 | 0.963 | 0.524 | 0.531 | 0 | Remain |
| Item 43 | 12.063^c^ | 0.640 ^b^ | 0.620 | 0.963 | 0.605 | 0.437 | 0 | Remain |
| Item 44 | 11.179^c^ | 0.654 ^b^ | 0.633 | 0.963 | 0.581 | 0.593 | 0 | Remain |
| Item 45 | 13.447^c^ | 0.665 ^b^ | 0.646 | 0.963 | 0.543 | 0.450 | 0 | Remain |
| Item 46 | 13.693^c^ | 0.716 ^b^ | 0.698 | 0.963 | 0.603 | 0.624 | 0 | Remain |
| Criteria | ≥3.000 | ≥0.400 | ≥0.400 | ≤0.981 | ≥0.200 | ≥0.400 |  |  |
| Note: ^c^ indicates *P* < .001, and ^b^ indicates *P* < .01. | | | | | | | | |
