# Supplementary material for: Digital Health Literacy Questionnaire for Older Adults: Instrument Development and Validation Study
Source: J Med Internet Res. 2025 Mar 19;27:e64193. doi: 10.2196/64193 (PMC11966078; doi:10.2196/64193)
Supplement: Multimedia Appendix 4 [file jmir_v27i1e64193_app4.docx]

**Multimedia Appendix 4:** Exploratory Factor Analysis factor loadings Matrix of the DHL Questionnaire for the older adults

| **Items** | **1** | **2** | **3** | **4** | **5** | **6** |
| --- | --- | --- | --- | --- | --- | --- |
| 1. I pay attention to whether health information is released and disseminated by official or authoritative institutions. | 0.694 |  |  |  |  |  |
| 2. I compare similar health information. | 0.670 |  |  |  |  |  |
| 3. I verify the correctness of health information from other sources. | 0.662 |  |  |  |  |  |
| 4. I remain vigilant about the health information I obtain and do not easily believe it. | 0.658 |  |  |  |  |  |
| 5. I understand digital health technologies (such as wearable devices, smart health electronic products, etc.) | 0.656 |  |  |  |  |  |
| 6. I understand that digital technologies can be used for health management or health promotion (such as health and medical mobile applications) | 0.654 |  |  |  |  |  |
| 7. I do not immediately share health information with others after receiving it but first check the content. | 0.648 |  |  |  |  |  |
| 8. I can browse, search, and obtain health information through digital devices or software. | 0.636 |  |  |  |  |  |
| 9. I pay attention to updates on health information | 0.635 |  |  |  |  |  |
| 10. I can judge whether health information is related to commercial interests (e.g., contains product advertisements) | 0.588 |  |  |  |  |  |
| 11. I understand that digital health devices or software can be used to store personal health information. | 0.574 |  |  |  |  |  |
| 12. I have used digital health devices or software to record personal health information. | 0.571 |  |  |  |  |  |
| 13. I know how to use digital health tools to track my health behavior. |  | 0.758 |  |  |  |  |
| 14. I can judge whether digital health tools are trustworthy. |  | 0.742 |  |  |  |  |
| 15. During the use of digital health tools, I can adjust my frequency, intensity, and methods based on the actual situation. |  | 0.703 |  |  |  |  |
| 16. I am used to using digital services to handle health information. |  | 0.701 |  |  |  |  |
| 17. I can use digital devices or electronic health products or software. |  | 0.686 |  |  |  |  |
| 18. I know when, how, and what health information to use. |  | 0.684 |  |  |  |  |
| 19. If necessary, I think I can persist in using digital health tools. |  | 0.647 |  |  |  |  |
| 20. I believe the use of digital technologies is beneficial for my health management. |  | 0.576 |  |  |  |  |
| 21. I believe I have the right to pursue legal responsibility for unauthorized data acquisition or improper data storage that leads to data breaches. |  |  | 0.770 |  |  |  |
| 22. I believe I have ownership of personal data, and others can only obtain my personal health data with my authorization . |  |  | 0.755 |  |  |  |
| 23. I do not click on unsafe web links; I do not visit websites that are flagged as risky. |  |  | 0.732 |  |  |  |
| 24. I can avoid health risks related to the use of digital technologies that threaten physical and mental health. |  |  | 0.676 |  |  |  |
| 25. I know the potential security risks in the online environment. |  |  | 0.674 |  |  |  |
| 26. I can share information with others on the internet. |  |  |  | 0.736 |  |  |
| 27. I can share information I obtained online with others offline. |  |  |  | 0.687 |  |  |
| 28. I can use digital devices or software to communicate health information with others. |  |  |  | 0.673 |  |  |
| 29. I am familiar with the user interface of digital devices or software. |  |  |  | 0.648 |  |  |
| 30. I use information dissemination platforms (such as Weibo, WeChat Moments, etc.) to share information. |  |  |  | 0.569 |  |  |
| 31. I can use digital devices or software to communicate health information with artificial intelligence (AI). |  |  |  | 0.556 |  |  |
| 32. I imitate the health-promoting behaviors or health management methods mentioned in health information. |  |  |  | 0.452 |  |  |
| 33. I can edit and improve health content created by myself or others. |  |  |  |  | 0.773 |  |
| 34. I can protect the integrity of original works and cite sources when referencing. |  |  |  |  | 0.757 |  |
| 35. I can integrate health information from multiple sources and rephrase it. |  |  |  |  | 0.747 |  |
| 36. In the past 12 months, I have participated in online health lectures and health care experience sharing activities. |  |  |  |  | 0.601 |  |
| 37. I adhere to correct political direction in online behavior. |  |  |  |  |  | 0.815 |
| 38. I do not fabricate or spread false, unverified health information. |  |  |  |  |  | 0.801 |
| 39. I care about health information related to myself. |  |  |  |  |  | 0.525 |
