# Supplementary material for: Digital Health Literacy Questionnaire for Older Adults: Instrument Development and Validation Study
Source: J Med Internet Res. 2025 Mar 19;27:e64193. doi: 10.2196/64193 (PMC11966078; doi:10.2196/64193)
Supplement: Multimedia Appendix 5 [file jmir_v27i1e64193_app5.docx]

**Multimedia Appendix 5:** Criterion-Related Validity of the DHL Questionnaire in older adults

|  | Information | Behavior | Safety | Interaction | Content | Attitude | DHL | Application Ability | Judgment Ability | Decision-Making Ability | eHEALS |
| --- | --- | --- | --- | --- | --- | --- | --- | --- | --- | --- | --- |
| Information | 1 |  |  |  |  |  |  |  |  |  |  |
| Behavior | 0.779^b^ | 1 |  |  |  |  |  |  |  |  |  |
| Safety | 0.614 ^b^ | 0.650 ^b^ | 1 |  |  |  |  |  |  |  |  |
| Interaction | 0.804 ^b^ | 0.773 ^b^ | 0.629 ^b^ | 1 |  |  |  |  |  |  |  |
| Content | 0.660 ^b^ | 0.704 ^b^ | 0.465 ^b^ | 0.708 ^b^ | 1 |  |  |  |  |  |  |
| Attitude | 0.555 ^b^ | 0.518 ^b^ | 0.676 ^b^ | 0.560 ^b^ | 0.363 ^b^ | 1 |  |  |  |  |  |
| DHL | 0.925 ^b^ | 0.904 ^b^ | 0.770 ^b^ | 0.908 ^b^ | 0.774 ^b^ | 0.671 ^b^ | 1 |  |  |  |  |
| Application Ability | 0.731 ^b^ | 0.858 ^b^ | 0.580 ^b^ | 0.778 ^b^ | 0.651 ^b^ | 0.456 ^b^ | 0.832 ^b^ | 1 |  |  |  |
| Judgment Ability | 0.675 ^b^ | 0.797 ^b^ | 0.538 ^b^ | 0.675 ^b^ | 0.660 ^b^ | 0.423 ^b^ | 0.768 ^b^ | 0.824 ^b^ | 1 |  |  |
| Decision-Making Ability | 0.640 ^b^ | 0.787 ^b^ | 0.538 ^b^ | 0.672 ^b^ | 0.631 ^b^ | 0.435 ^b^ | 0.750 ^b^ | 0.816 ^b^ | 0.825 ^b^ | 1 |  |
| eHEALS | 0.742 ^b^ | 0.877 ^b^ | 0.593 ^b^ | 0.777 ^b^ | 0.684 ^b^ | 0.468 ^b^ | 0.847 ^b^ | 0.980 ^b^ | 0.913 ^b^ | 0.886 ^b^ | 1 |

Note: ^b^ indicates *P* < .01.
